# Systematic biomedical research of the NASA Twins Study facilitates the hazard risk assessment of long-term spaceflight missions

**DOI:** 10.1007/s13238-019-0628-x

**Published:** 2019-05-20

**Authors:** Zhongquan Dai, Xiaohua Lei, Chao Yang, Lei Zhao, Liang Lu, Yinghui Li

**Affiliations:** 1 State Key Laboratory of Space Medicine Fundamentals and Application, China Astronaut Research and Training Center, Beijing, 100094 China; 2 State Key Laboratory of Stem Cell and Reproductive Biology, Institute of Zoology, Chinese Academy of Sciences, Beijing, 100101 China; 3 Institute of Environmental Systems Biology, College of Environmental Science and Engineering, Dalian Maritime University, Dalian, 116026 China

With the extension of the deep space exploration program, the boundaries of human exploration will be pushed forward to the surface of Mars. Over the past nearly 60 years manned spaceflight and experimental findings demonstrated that spaceflight induced bone loss, muscle atrophy, cardiovascular remodeling and space-related medical problems (Durante and Cucinotta, 2008). There are some long-term spaceflight missions planned to implement in the next decades, however, health risks and protections from spaceflight exposures are incompletely clear and still remain a primary concern for manned deep space explorations (Zhao et al., 2018; Wang et al., 2019). Although more than 560 people have the spaceflight experience, only four individuals have participated in long-term spaceflight missions lasting 1 year or more (Stepanek et al., 2019). There is an urgent need to better understand the hazards of long-term spaceflight environment, including weightlessness, ionizing radiation, confinement, disrupted circadian rhythm etc.

Recently, one work published in Science (Garrett-Bakelman et al., 2019) showed a systematic biomedical research of the NASA Twins Study and health risk assessments during a 1-year mission onboard the International Space Station (ISS). The use of twin model is an excellent experiment design with the improved reliability and authenticity of the obtained data from this small sample experiment. As compared to his twin on the earth, the astronaut living in space presented some changes in telomeres, DNA damage and methylation, immune, microbial, mitochondrial, cardiovascular, body mass and nutrition, neuro-ocular, cognitive performance. First, the authors found that the telomere unexpectedly elongated during spaceflight and distribution toward increased numbers of longer telomeres, which was soon shortened after returning to the earth. And, the increased average telomere length was in accordance with the methylation changes of TERT promoter, the regulatory subunit of telomerase, supported by gene expression, correlated with reduced body mass and increased serum folate levels inflight. However, it is still unknown whether the elongated telomeres imply a higher risk of disease occurrence (Helby et al., 2017). Chromosome aberrations were also analyzed to evaluate potential telomere-related instability and DNA damage response to ionizing radiation exposures, and the results showed that the inversion frequencies of inflight twin increased at a greater rate than translocations, which are consistent with the physical dose of 76.18 mSv and an effective dose of 146.34 mSv recorded by NASA. Moreover, subtle alternations in the global and local DNA methylation levels and entropies discordances were observed in CD4 and CD8 lymphocytes by the methods including the principal components analysis and genome-wide JSD analysis, etc., and the genes according to the epigenetic discordances were therefore ranked and conducted by the gene ontology enrichment analysis. Furthermore, differential gene expression analysis and cytokine data of inflammation signatures clearly showed many immune-related pathways were significantly influenced and inflammatory status was increased significantly during inflight. This adds the weights to support that the space radiation and weightlessness in spaceflight can induce the significant changes in the DNA damage and inflammation (Sridharan et al., 2015; Chakraborty et al., 2018).

It was of interesting to find out that a series of mitochondria-related changes were identified by integrated analysis, including increased basal respiration and decrease of ATP reserve of plasma, higher level of lactic acid indicating a shift from aerobic to anaerobic metabolism. Previous works also suggested that energy imbalances potentially lead to changes with protein metabolism that ultimately impair the immune system (Chakraborty et al., 2014). In accordance with previous NASA reports, recent evidences showed 29% visual impairment of short-term spaceflight astronaut and 60% of long-duration mission crew (Marshall-Goebel et al., 2017). Spaceflight resulted in ocular change and retinal edema formation of spaceflight twin, including shape change, thickened retinal nerve, folds appeared in the choroid layer, but not occur in ground control. This space-associated neuro-ocular syndrome (SANS) was caused by fluid headward shift (Lee et al., 2018), which also leads to the distension of the neck’s jugular vein, more cardiac output and a thickening of the forehead’s skin.

**Figure 1:**
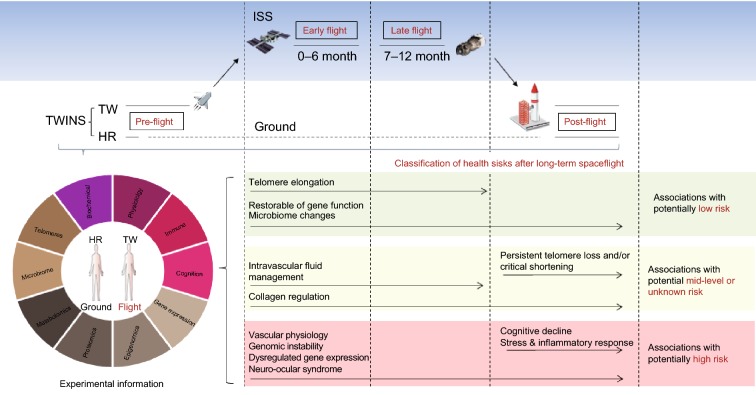
**Systematic biomedical research of the NASA Twins Study**. Study design, experimental information and classification of the putative health risks for future human spaceflight. This study lasted over 25 months, including time points before (preflight), 0–6 month flight (early flight), 7–12 month flight (late flight) and after (postflight) spaceflight. TW (flight subject), HR (ground subject). This risk classification is of great significance for the disease risk estimations of astronauts in spaceflight

Finally, the researchers systematically classified the influenced key physiological processes into 1) potentially low risk, 2) moderate risk and unknown risk, 3) potentially high risk. The SASN, cardiovascular physiology, postflight stress and inflammatory response, genomic instability and dysregulated gene expression were considered as potentially high risks (Fig. 1). In general, such a risk classification is of great significance for the disease risk estimations of astronauts in spaceflight, however further analyses by the quantitative methods are needed to assess the health risk accurately for deep space exploration missions.

Taken together, this twin spaceflight study embodied an investigation trend to deeply evaluate the risk of more than 1-year long-term manned spaceflight: 1) Systematic multidimension analysis; 2) Multicenter joint analysis; 3) Interactive verification analysis of multi-data to improve the reliability and authenticity because of small samples; 4) Mutual regulation among organs or tissues; 5) Energy metabolism variation analysis. However, there are large challenges on the integration analysis of the multidimensional data with the features of limit individuals and time series. Therefore, more effective data-mine methods are needed for the analysis of these complex datasets. Nevertheless, this study provides a theoretical and data reference for the subsequent study of human space flight, and is of landmark significance in the field of space medicine.
